# Reconstructing the Antibiotic Pipeline: Natural Alternatives to Antibacterial Agents

**DOI:** 10.3390/biom15081182

**Published:** 2025-08-18

**Authors:** Chiemerie T. Ekwueme, Ifeoma V. Anyiam, David C. Ekwueme, Christian K. Anumudu, Helen Onyeaka

**Affiliations:** 1 School of Health and Life Sciences, Teesside University, Middlesborough TS1 3BX, UK; theresaekwueme0622@gmail.com (C.T.E.); davebrain.dc@gmail.com (D.C.E.); 2 Department of Microbiology, Faculty of Science, Federal University Otuoke, Otuoke 562103, BY, Nigeria; cka329@student.bham.ac.uk; 3 School of Chemical Engineering, University of Birmingham, Edgbaston B15 2TT, UK

**Keywords:** antimicrobial resistance, natural alternatives, antibacterial pipeline, natural products, drug discovery

## Abstract

The discovery of penicillin led to remarkable progress in the treatment of diseases and far-reaching advancements in novel antibiotics’ development and use. However, the uncontrolled use and abuse of antibiotics in subsequent years have led to the emergence of the antimicrobial resistance (AMR) crisis, which now threatens modern medicine. There is an increasing number of emerging and reemerging infectious diseases, which have worsened the state of AMR and pose a serious threat to global health. The World Health Organization (WHO) reports the inadequacy of the drug development pipeline to meet the needs of the pharmaceutical sector in the face of AMR, and this poses a significant challenge in the treatment of diseases. Natural products (NPs) represent a promising group of antibiotic alternatives that can potentially mitigate AMR, as they bypass the pharmacodynamics of traditional antibiotics, thereby making them immune to the mechanisms of AMR. NPs, including plant derivatives, bacteriophages, metals, antimicrobial peptides, enzymes, and immune modulators, as monotherapies or in synergism with existing antibiotics, are gaining attention in a bid to reconstruct the antibiotic pipeline. Harnessing these as antimicrobial agents to curb AMR can help to provide sufficient defence against these infectious pathogens. The current review provides a comprehensive overview of the state of AMR and the potential of the above-mentioned antibiotic alternatives. Additionally, we discuss progress made and research breakthroughs in the application of these alternative therapies in humans, exploring findings from clinical trials and experimental models. The review further evaluates the advancement in technology, interdisciplinary approaches to the formulation and utilisation of NPs, and collaborations in alternative drug development. The research gaps present in this ever-evolving field are highlighted and evaluated together with regulatory issues, safety concerns, and technical difficulties in implementation.

## 1. Introduction

Infectious diseases have been a leading cause of mortality among humans and animals. In the not-so-distant past, infectious microbial diseases were almost incurable. Diseases such as smallpox and cholera ravaged the world, and it is estimated that the bubonic plague, caused by *Yersinia* sp., resulted in the death of more than one-third of the population of Europe in the 14th century [1,2]. During this age, although the knowledge of disease prognosis and accurate diagnosis was limited, several agents, including poultices, mould, and plant derivatives, were used to treat infectious diseases with varying approaches and success rates [3]. The accidental and unprecedented discovery of the antibiotic penicillin by Alexander Fleming was a major breakthrough in mitigating the problem of infectious diseases [4]. This brought about the “golden age” of antibiotics, which drastically changed contemporary medicine, revolutionising the treatment of infectious diseases, including previously deadly bacterial infections [5,6]. It further bridged the gap between previous traditional methods of treatment and targeted use of chemical agents for disease treatment [6]. Subsequently, remarkable progress was made in discovering new products effective against microorganisms, including macrolides, third-generation cephalosporins, and semi-synthetic antibiotics like amoxicillin and some quinolones, which are effective against a relatively wider spectrum of microorganisms, marking the peak of the antibiotic age [7,8,9]. However, decades after the mainstreaming of antibiotics in clinical practice and following widespread access, infectious diseases have again become a problem in patient care due to the evolutionary response of infectious pathogens to treatment and the development of resistance [10].

The emergence of antimicrobial resistance (AMR) threatens the success of antibiotic therapy and limits existing treatment options. The growing problem of AMR has been a burden on the global healthcare system over the past few decades and is linked to significantly high rates of morbidity and mortality. In 2019, AMR directly led to 1.27 million deaths and was implicated in a further 4.95 million deaths [11]. AMR has further led to treatment failures in specific disease conditions due to reduced drug efficacy. In 2020, one in five cases of urinary tract infection (UTI) caused by *Escherichia coli* reportedly showed reduced susceptibility to standard antibiotics [12]. It is estimated that with the current trend, by 2050, AMR-related deaths will rise to 10 million, in the absence of intervention [13].

Several studies have reported the indiscriminate use of antibiotics and their environmental exposure to be the major precipitator of bacteria’s evolutionary response to antibiotics, leading to the development of resistant strains [14,15]. Despite the far-reaching consequences of the ever-worsening AMR problem, the crisis is being poorly handled in the areas of regulatory control and new-drug rollout. Amidst the rapid occurrence of resistance to conventional antibiotics, the drug development pipeline is slow to match this, with an average time of about 8 years for a drug to be developed from phase 1 clinical trials to product launch [16]. The WHO reports that only 13 new antibacterial agents have been developed since 2017, with only 2 representing a new chemical class [17]. Overall, the timeframe required for developing these drugs and conducting clinical trials, the likelihood of failure, and the lack of innovation threaten the future of clinical interventions [17]. Previously, medical research stayed ahead of AMR, but a decline has been reported; hence, we are presently faced with a scenario where it is projected that, by 2050, the world will run out of antimicrobial agents for treating diseases [18,19]. In the last few years, the development of drug options from natural products (NPs) has been at the forefront of research to eliminate potential deleterious effects associated with common synthetic drugs and bacterial response to natural antimicrobials [20].

Natural alternatives (NAs) to antimicrobial agents, including “natural products”, are now considered the potential silver bullets. Vaccines, phytochemicals, and other plant-based products, metals, microbiome-based interventions, bacteriophages, immune-modulating agents, peptides, and antimicrobial enzymes, amongst others, are increasingly being explored to complement treatments with antimicrobials and possibly replace them [17,21,22,23,24]. These alternatives offer advantages over traditional antimicrobials due to the potential for novel mechanisms of action, which can either slow down or bypass microorganisms’ ability to develop resistance. They also offer promises in broad-spectrum bioactivity and synergistic effects that enhance the efficacy of existing antimicrobials, thus ensuring sustainability in the long term [23]. Furthermore, the discovery of new antibiotics is technically demanding and expensive with low sales volume [25]. NPs can be cheaper and more readily synthesised alternatives to conventional antibiotics. The potential of NAs has been reported over the years, but with limited attention to a holistic and comparative approach to exploring the future of these diverse antibiotic alternatives. This study focuses on exploring the potential of NAs to antibiotics, which aims to minimise the effect of AMR and the potential deleterious effects associated with the use of conventional treatment options. It further delves into providing a substantial amount of evidence based on previous research to somewhat compare the degree of efficacy associated with prescribed antibiotics and alternative options, hence establishing the possibility of mainstreaming NAs to curb the problem of AMR, reconstruct the antibiotic pipeline, and provide new technologies to tackle infectious pathogens. This study systematically explores current progress in the utilisation of NAs, the future of this emerging class of therapeutics, current challenges, and possible gaps in research on different NAs, using defined focus areas and study subjects.

## 2. Antibiotics and the Emergence of AMR

Since the early days of recorded history, natural products (NPs) have been employed in the treatment of infectious diseases, such as the Egyptians’ use of mouldy bread and medicinal soils in treating infections [26], as outlined in an ancient document of Egyptian herbal knowledge called the *Ebers Papyrus* (1500 BC). Different civilisations have utilised different NPs in infectious disease control, until the 20th century saw the serendipitous discovery of penicillin by Alexander Fleming, which launched the “golden era” of antibiotics [4,7]. Following this discovery, remarkable progress was made in the development of several antibiotics for the treatment of infections. The post-World War II period brought about advances in the development of new antibiotics and bioactive compounds, including semi-synthetic antibiotics, due to the associated stability and broad spectrum of activity of the newer classes of antibiotics, including the macrolides, third-generation cephalosporins, daptomycin, and linezolid [7].

The different classes of antibiotics have different mechanisms for exerting toxicity on pathogens; hence, they are classified based on their mechanisms of action and chemical structures [27]. These can include cell wall synthesis inhibition (e.g., carboxypeptidase), protein biosynthesis inhibition (e.g., aminoglycosides), interference with DNA replication by targeting DNA gyrase and topoisomerase IV (e.g., fluoroquinolones), and inhibition of folic acid metabolism (e.g., sulfonamide) [28]. Generally, antibiotics target and eliminate infectious bacterial pathogens or inhibit their growth in the body through any or a combination of these complex metabolic pathways. In simple terms, this may involve the oral consumption of antibiotics, followed by their absorption through the gastrointestinal tract and distribution to infection sites, or delivery through other means, including intravenous administration, intramuscular administration, etc. Upon their absorption and arrival in the invading microbes’ environment, they exert a toxic effect, which leads to the elimination of pathogens, thus treating the infection. However, there is usually an incomplete metabolism of antibiotics, and thus, antibiotic residues and unmetabolised antibiotics are excreted and enter the environment [29]. The emergence of AMR is linked to the indiscriminate use of antibiotics, which has led to the selection of resistant bacteria and horizontal gene transfer (HGT) among bacteria and now threatens their use in patient care [30]. AMR dates to the discovery of antibiotics and their application in clinical use. This phenomenon describes the heritable ability of bacteria to persist in the presence of a high concentration of antibiotics [31]. The idea of AMR is mostly mistaken for bacterial tolerance, which describes a bacterium’s ability to survive an increased antibiotic concentration within its minimum inhibitory concentration (MIC) and is usually conferred by environmental stress conditions [32]. Although both are mechanisms by which microbes survive antimicrobial treatment, they usually differ in their underlying biology and clinical implications. AMR is usually heritable, and this resistance is typically due to genetic mutations or acquisition of resistance genes, and this usually leads to therapeutic failure, as the microbe can continue to replicate despite the presence of the antimicrobial agent/drug at standard concentrations. In contrast, antimicrobial tolerance is typically non-heritable and does not involve the ability of the microbe to grow in the presence of the drug, but rather its ability to survive transient exposure without necessarily multiplying. Within the scope of this study, we will use the term AMR to describe any form of antibiotic treatment failure. AMR is caused mainly by four survival mechanisms adopted by bacteria; these abilities may be native or acquired [33] and include efflux pump mechanisms, modification of molecular targets, enzymatic inactivation, and limitation of drug uptake.

### 2.1. Efflux Pump Mechanism

Bacteria possess a plethora of efflux pumps (transport proteins) located in the plasma membrane that function by transporting molecules outside cells, including toxic substances, quorum-sensing molecules, and antibiotics. Evidence suggests that the primary role of these pumps is not extruding antibiotics [34,35]. Nonetheless, certain pumps such as *TetA* and *CmlA* have been shown to exert specific intrinsic resistance to antibiotics [36]. Upon exposure to antibiotics, bacteria, in many cases, over-express these efflux pumps, resulting in a low intracellular level of antibiotics, impairing the ability of the antibiotics to reach the intended target [37]. Gaurav et al. [38] describe bacterial efflux pump systems and how they confer AMR in detail.

### 2.2. Modification of Molecular Target

Antibiotics are designed to target specific molecules within a bacterium to eliminate it; however, certain bacteria, by modifying their ribosomal 30S or 50S subunits, change their structure and prevent antibiotic functions [39]. For example, penicillin-binding proteins (PBPs) have been shown to play a vital role in the biosynthesis of bacterial cell walls and are the primary targets of β-lactam antibiotics [40]. A PBP modification was reported by Kuo et al. [41] and linked to amoxicillin resistance in *Helicobacter pylori*. Similarly, AMR in *Streptococcus pneumoniae* has been linked to modification of associated PBP [42]. Several other modifications of molecular targets and AMR have been reported [28].

### 2.3. Enzymatic Inactivation of Antibiotics

Certain enzymes within a bacterium protect the cells from toxic substances by modifying or degrading antibiotics, thereby inactivating them. Hydrolases, transferases, and oxidoreductases are known to contribute to the inactivation of antibiotics. A group of β-Lactamases efficiently hydrolyse cephalosporins [43], thus rendering them inactive. Furthermore, resistance to certain macrolides, such as erythromycin and azithromycin, is due to the production of esterase, catalysing hydrolysis of the lactone ring [44]. Gjonbalaj et al. [45] showed that the expression of resistance genes by commensal bacterial species mediates the shielding of pathogens from antibiotics by degrading the antibiotics.

### 2.4. Limiting Drug Uptake

Lipopolysaccharide (LPS), which is a constituent part of Gram-negative bacteria, limits the access of certain antibiotics to the cell and makes them intrinsically less permeable to certain antibiotics. Naturally, certain bacteria lacking cell walls are inherently resistant to antibiotics that disrupt cell wall components. However, Gram-negative bacteria possess porin channels on their outer membrane that allow hydrophilic molecules to enter the cell [46], thus providing a pathway for antibiotics to enter and act on the organism. However, these porins may undergo modifications that change the selectivity of the porin channel and/or decrease the number of porins present [47]. OmpC (a type of porin) has been reported to facilitate the absorption and resistance of antibiotics, such as β-lactams. Similarly, Tina et al. [48] suggest that the key player in carbapenem resistance is a change in the amino acid configuration in the ompC of carbapenem-resistant *E. coli*. Another porin, OmpF, which plays a significant role in the permeation of antimicrobial peptides (AMPs), has been reported to be the primary pathway for antibiotic entry [49]. Among different bacteria, the ompF mutant has been found to be resistant to many β-lactam antibiotics [50]. Another major factor that limits drug uptake is the formation of biofilms. About 99% of bacterial species can produce biofilm, which is a structured community of microorganisms enclosed in a complex self-produced surface-bound extracellular polymeric substance (EPS) [51]. These surfaces could be either living (e.g., skin and mucosa of the human body) or non-living (e.g., artificial knee joints and venous catheters). Biofilms limit antibiotic penetration and reduce bacterial metabolic rates, rendering many antibiotics ineffective [52,53]. Within biofilms, bacteria exhibit up to 1000-fold higher resistance to antibiotics compared to their planktonic counterparts, owing to the presence of thick EPS layers, persistent cells, and enhanced expression of efflux pumps [54].

Biofilm development is tightly regulated by quorum sensing (QS), which is a complex mode of communication between bacteria. QS enables bacterial communities to synchronise their behaviour in response to changes in cell population density and the composition of nearby microbial communities [55]. This process primarily occurs via QS molecules such as Acyl homoserine lactones (AHLs), autoinducer-2 (AI-2), and autoinducing peptides (AIPs) [56,57]. Although specific mechanisms vary between species and environments, biofilm formation typically involves the reversible and irreversible attachment of bacteria to a surface and microcolony formation, involving EPS production, maturation, and detachment [56]. Upon biofilm formation, bacteria can respond to environmental cues and recruit co-aggregating species that support biofilm maturation. For example, *Hoylesella loescheii* (formerly *Prevotella loescheii*) can produce proteases that hydrolyse its adhesins and recruit *Streptococcus mitis* [58]. Following maturation, cells may detach from the substrate as planktonic cells contributing to infection dissemination [59].

Notably, the ESKAPE pathogens (*Enterococcus faecium*, *Staphylococcus aureus*, *Klebsiella Pneumoniae*, *Acinetobacter baumannii*, *Pseudomonas aeruginosa*, *Enterobacter* sp.), described as a highly resistant group of bacteria and a current global healthcare-associated infection focus, have been reported to adopt all mechanisms of action of antimicrobial resistance described above [60]. Based on these bacterial mechanisms for evading antibiotics, the chances of AMR remaining a threat to public health are high. Thus, the best hope for treating infectious diseases lies in the use of alternatives, including NPs due to their unrivalled chemical diversity and potential to bypass bacterial mechanisms against antibiotics.

## 3. The State of Global AMR

The problem of AMR is growing every year. In 2019, untreatable bacterial infections were linked to an estimated 4.95 million deaths, of which 1.27 million deaths were attributable to bacterial AMR. Of these, methicillin-resistant *Staphylococcus aureus* (MRSA) resulted in over 100,000 deaths the same year [61]. These figures exceed the reported annual deaths caused by tuberculosis (1.5 million), malaria (643,000), and HIV/AIDS (864,000) [62]. A review of AMR reported by O’Neill [13] projected that AMR could kill 10 million people every year by 2050. Furthermore, the World Health Organization (WHO) has recognised AMR as an urgent public health threat [63,64]. A study by Mestrovic et al. [65] assessed the prevalence of AMR in the WHO European region, estimating a total burden of 541,000 deaths due to bacterial AMR, with the numbers for 2019 alone estimated at 133,000 deaths. The largest burden was found to be from bloodstream infections (195,000 deaths), followed by intra-abdominal infections (127,000 deaths). MRSA was reported as the leading pathogen responsible for associated deaths in 27 countries within the region. Data from the European Union Summary Report on Antimicrobial Resistance in Zoonotic and Indicator Bacteria from Humans, Animals and Food in 2021–2022 indicates high to extreme levels of resistance to antibiotic therapy [66]. These figures are expected to grow higher without intervention, which would severely impact public health. Unfortunately, the bulk of the global impact of AMR is borne by low-income and middle-income countries (LMICs) [61]. In a recent report focused on Africa, 1.05 million deaths were linked to bacterial AMR, and 250,000 deaths were attributable to bacterial AMR in 2019. *S. pneumoniae*, *Klebsiella pneumoniae*, *E. coli*, and *S. aureus* were the leading causes of death, implicated in over 100,000 deaths each. Of the deaths attributable to AMR, 53% and 34% were linked to third-generation cephalosporin-resistant *K. pneumoniae* and MRSA, respectively [67]. The Global Antimicrobial Resistance and Use Surveillance System (GLASS) report (2022) highlights a 15% increase in AMR rates among common drug options in 2020 compared to 2017 [68]. Median AMR rates of 42% for third-generation cephalosporin-resistant *E. coli* and 35% for MRSA have been reported as a serious concern [12]. Overall, there is an alarming increase in global AMR that threatens public health. Importantly, while the consequences of AMR for human health have been reported, data on the current burden of AMR and impact on animals remain scarce [69].

The development of AMR has impacted the global market for antibiotics, especially around the over-consumption of existing antibiotics, lack of new drug discovery, and under-production of new antibiotics [70]. The World Bank estimates an increase of USD 1 trillion in healthcare costs due to AMR and a reduction of 1.1–3.8% in global domestic product per year by 2050, with an estimated impact of USD 9 billion per year. Again, the gross domestic product (GDP) losses per year are expected to increase by over 240% in 2030 [71,72,73,74]. The World Bank report also highlights that AMR may likely impact agriculture, leading to lower livestock production due to a greater burden of untreatable infectious diseases in livestock [74]. This problem is exacerbated by a lack of progress in AMR research, with the WHO reporting a correlation between the inadequacy of antimicrobial drug research and drug rollout with the rise of AMR [12]. This poses a significant challenge in the treatment of diseases, hence the need to improve the drug discovery pipeline with alternative treatment options.

While national and global efforts in AMR surveillance have provided valuable insights into AMR and approaches to address its spread, these surveillance systems, such as GLASS, face several limitations that constrain their effectiveness. One of such key limitations is the variation in national laboratory and surveillance capacities, particularly between high-income and low- and middle-income countries. Many participating countries lack the infrastructure, technical expertise, or financial resources required for consistent, high-quality data collection and reporting [75]. This results in incomplete and uneven data coverage, limiting the comparability of data across regions and potentially underestimating the true burden of AMR globally. Another critical issue is the limited scope of pathogens and antimicrobials included in many of these reports, which focus primarily on a narrow set of priority pathogens. This narrow focus excludes resistance data on many clinically relevant microorganisms, such as fungi and emerging resistant organisms, reducing the system’s comprehensiveness. Moreover, systems such as GLASS rely heavily on passive data collection, which may disproportionately represent data from tertiary care hospitals, urban centres, or more well-resourced laboratories while underrepresenting rural and primary healthcare settings, where surveillance is often weakest [76]. Finally, political, legal, and logistical barriers can hinder timely data sharing and transparency, as some countries may be reluctant to report high resistance rates for fear of economic or reputational consequences [75]. Despite these limitations, global AMR surveillance remains a critical tool in the fight against AMR, and addressing the shortcomings of these systems, especially at the sub-national levels through capacity building, investment in laboratory infrastructure, and stronger international collaboration, is essential for their continued development and effectiveness.

## 4. Natural Alternatives to Antibacterial Agents

**Figure 1:**
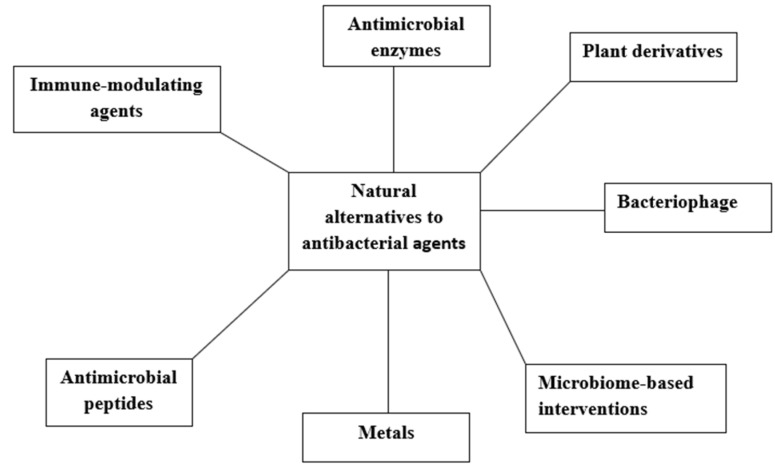
Natural alternatives to antibacterial agents.

Within the last decade, non-traditional biological agents have represented an active and innovative preclinical pipeline with an effective strategy to curb AMR. These treatment options trace back to ancient history, which placed NPs at the heart of medicine. Traditional folk remedies and herbal medicines originated from mostly plant derivatives and are based on medical recipes that have played major roles in modern medicine. Generally, NPs are reservoirs for biological compounds that can be harnessed as antibacterial agents to curb AMR and provide sufficient defence against infectious pathogens. Plant derivatives, bacteriophages, metals, antimicrobial peptides, enzymes, and immune modulators are NPs that are gaining attention in a bid to reconstruct the antibacterial drug pipeline (Figure 1).

### 4.1. Some Antibacterial Alternatives of Natural Origin

#### 4.1.1. Plant Derivatives

Archaeological findings suggest that the use of medicinal herbs dates to 60,000 years ago [77,78]. The Palaeolithic era began some 3.4 million years ago with the emergence of stone tools, and during this time, people likely used plant-derived medicines to treat ailments [79,80]. In the early modern age, plants and their derivatives were employed in the treatment of diverse illnesses, some of which were of microbial origin. With the emergence of many deadly epidemics in this age, medicinal plants were curated to be utilised as therapeutics, with antibacterial compounds within plants actively being explored for therapeutic uses long before the antibiotic era [81]. The oldest scripted evidence of the use of medicinal plants in the preparation of medicines dates to writings of the Sumerians approximately 5000 years ago, discovered on a Sumerian clay slab from Nagpur that contained recipes for medicine preparations [77]. Similarly, ancient Chinese writing on roots and grasses (2500 BC) highlights 365 drugs, some of which are currently being used. Again, the *Ebers Papyrus* (1500 BC) reports a collection of 800 prescriptions involving the use of 700 plants. The works of Hippocrates (459–370 BC) classified 300 medicinal plants based on their physiological action. An Indian holy book, the *Bible*, and holy Jewish writings, amongst others, have also reported the use of medicinal plants in the past [82]. The antibiotic era diverted significant attention to natural antibiotics to cure bacterial infections and soon led to the production of synthetic antibiotics. However, in the present age, plant-derived medicine is still the mainstay of about 80% of the world population; meanwhile, only 16% of plants have been tested for biological activity [79,83].

Plants act therapeutically through several mechanisms and help to restore the physiological balance of a patient’s body, enhancing resistance to infectious pathogens, and can serve as a synergetic tool in disease control [81,84]. The mode of action of plant antibacterial compounds is mainly attributed to the inhibitory effect of the plant’s phytochemical compounds. Khameneh et al. [85] reported that the chemical interference of phytochemicals with the biosynthetic processes and metabolism of bacteria is the primary mechanism of their antibacterial activity. Major groups of phytochemicals include alkaloids, sulfur-containing compounds, terpenoids, and polyphenols with primary metabolic properties. In a recent study, Alemu et al. [86] undertook phytochemical studies and assessed the antibacterial activity of five medicinal plants in Ethiopia against *S. aureus*, *Escherichia coli*, and *Streptococcus agalactiae*. The results showed the presence of steroids, alkaloids, flavonoids, saponins, and terpenoids, which exerted broad-spectrum activity against selected isolates. In another study, galantamine from *Rauhia multiflora* was also reported as a major alkaloid suppressing antibacterial activity [87]. Similarly, mucilage, essential oils, fixed oils, fat-soluble vitamins, sterols, and waxes are secondary metabolites that have also been reported in antibacterial studies. In another recently published paper, Mangalagiri et al. [88] assessed the antibacterial effect of essential oils against nonclinical non-pathogenic bacteria, pathogenic bacteria, and MDR strains. Antibacterial activity was observed across all bacterial groups, with killing times comparable to those of marketed antibiotics. For example, the essential oil of lemon grass showed 100% inhibition of *E. coli* at 90 min, which is similar to that of ampicillin. Again, Taibi et al. [89] assessed the antibacterial activity of *Ptychotis verticillata* essential oil (PVEO) and its synergetic activity with antibiotics using the Fractional Inhibitory Concentration Index (FICI). Although antibacterial properties were observed against isolates, a high FICI was observed between PVEO and amoxicillin, with a significant reduction ranging from two- to sevenfold. This highlights that plant derivatives are promising as single-channel antibacterial agents as well as synergetic antibacterial agents in the treatment of MDR pathogens. Several other studies have reported progress in the assessment of plant-derived chemical compounds on culture collections and clinical and nonclinical bacterial isolates (Table 1). Although the studies in Table 1 report the MICs in mg/mL and the question of the final concentrations that can reach the systemic circulation is therefore raised, there is substantial evidence suggesting that plant-derived compounds are effective in the treatment of bacterial infectious diseases based on these studies. Also, the capital and scientific rigour required to achieve clinically administrable doses is a big challenge, which has limited the drive to take a medicinal-plant-based drug from bench to bedside, which has limited research in the past. However, recently, more effort is being made around this promising field of research.

#### 4.1.2. Bacteriophages

Bacteriophages, or phages/bacteria-eaters, are a promising treatment option for several bacterial infections. They are viruses with a size range of 20–200 nm that infect bacteria and are classified based on their size and morphological characteristics. They possess tails, but some may be filamentous and pleomorphic [100]. Their virions are composed of double-stranded or single-stranded DNA or RNA and a protein coat made of lipid envelopes. Based on infectivity, phages may be lytic or temperate, with the former causing cell lysis and the release of progeny phages and the latter involving the possibility of lytic and lysogenic growth pathways [100]. The use of bacteriophages therapeutically is currently limited by a low number of, and overly complex, clinical trials, which are further impacted by regulatory guidance/standards impeding their progress [101,102]. Phages are inherent and abundant members of the environmental and gut microbiomes and act by targeting specific bacteria [103]. The bacteria specificity associated with phage therapy allows phages to narrowly attack bacterial strains of interest by interfering with bacterial metabolism and activating lysis without altering the balance of the microbiota, in contrast to antibiotics that decimate the microbiota and, in an attempt to control the growth of one infectious pathogen, may trigger dysbiosis.

Early clinical studies on phage therapy received minimal attention in the scientific communities in most regions but were vigorously pursued within the Soviet Union and Eastern Europe. This resulted in most research reports being published in non-English journals, and hence, they were not widely accessible to Western scientists. Phage therapy dates back over a century. Before the work of Félix d’Herelle in 1896, Ernest Hankin, an English bacteriologist, discovered an inhibitory effect against *Vibrio cholerae* in water samples obtained from the Ganges and Yamuna Rivers in India. He suggested that this occurrence may have influenced the cutback during the cholera pandemic of 1881–1896 in the Ganges Delta, West Bengal [104,105]. Two years later, during the outbreak of haemorrhagic dysentery, Félix d’Herelle conducted research using faecal samples of hospitalised French soldiers and suggested from his experiments that the patches that appeared on his agar plates were due to a virus parasitising the bacterium. He went on to coin the term “bacteriophages” and made the first attempt at phage therapy [106]. However, controversies arose from previous narratives that questioned the longevity of this therapy, and with the advent of antibiotics, investigations into the use of bacteriophages as therapeutic agents in the Western world were dampened. Nonetheless, over the past decade, due to the theoretical comparative advantage of bacteriophages over antibiotics, especially in connection with increased levels of AMR, and several successes in individual case reports, phage therapy has gained in popularity [107]. Bacteriophages are specific. This means that it is uncommon for them to infect most strains within one species [108]. The phage specificity hurdle has been addressed by the *preˆt-a`-porter* approach, which attempts to create a broad-spectrum effect using a phage cocktail and *a la carte* phage therapy concept, which involves the use of a stored phage and a wild phage to target infecting bacteria [109]. The pharmacokinetics and pharmacodynamics of phage therapy (PT) rely on the in vitro ability of phages to multiply within target bacterial cells and trigger lysis upon reaching the infection site, which destroys the infectious cells but does not disrupt the microbiota. This lysis is accompanied by a reduction in the phage titres and complete elimination of both infectious cells and phages alike [110,111]. The available literature on phage therapy’s pharmacological data suggests that phages enter the bloodstream [112]; however, no trace was observed in the Göttingen minipigs used by Gencay et al. [113] to study the tolerability and gastrointestinal recovery of engineered phages. However, in other studies, phages have been found in several body organs of animal models, including the liver, gut, bone marrow, heart, thymus, etc. [114,115]. The adoption of phage therapy previously lagged due to the problem of immunogenicity, which involves the triggering of the immune system upon the entrance of the phage for the first time and the memory cells that trigger immune defence upon a subsequent entrance. However, Majewska et al. [116] demonstrated that phage immunogenicity in mice is significantly low. Additionally, advances in genetic engineering have now limited this immunogenicity problem, resulting in significant progress in this research area [117]. In several instances, PT administered via injection, immersion, or oral feeding has been successful in the treatment of bacterial infections, such as in a study that used PT to treat *Aeromonas hydrophilia* in animal models [118]. Table 2 presents a list of several case reports/studies using PT successfully in the treatment of ailments. However, the absorption, biodistribution, metabolism, and elimination of bacteriophages still pose limitations in their adoption in both clinical trials and therapeutic use [100,110].

**Table 2:** Representative case reports and case studies highlighting the antibacterial effects of bacteriophage therapy.

Bacteriophage	Report Type	Target Bacteria	Administration Route	Result	Reference
ɸ9184	Case report	*Enterococcus faecium*	Intravenous and oral adjuvant with daptomycin and vancomycin	Clinical improvement within 24 h is indicated by bacterial growth suppression.	[119]
Intestine bacteriophage cocktail	Case report	MDR (*P. aeruginosa*, *E. coli*, *K. pneumoniae*, *P. mirabilis*) and vancomycin-resistant *E. faecium*	Transdermal using hydrogel	Successful treatment of fracture-related infections with no need for surgical revision after a year.	[120]
14/10, PT07 and PNM *P. aeruginosa* bacteriophage	Case report	*P. aeruginosa*	Intravenous administration with ceftazidime–avibactam	A new *P. aeruginosa* infection occurred upon completion of phage therapy. Biofilm and phage-resistant mutants indicate possible antibiotic-influenced stress.	[121]
Phage-Muddy Muddy_HRM^GD04^	Case report	*Mycobacterium chelonae*	Intravenous adjuvant	Host immune reaction with stable improvement of the cutaneous infection.	[122]
D29_HRM^GD40^ BPsΔ33HTH_HRM10	Case study	*Mycobacterium abscessus*	Intravenous administration	Typical decline in bacterial diversity and no observed increase in resistance to phage therapy or antibiotics.	[123]
Different phage therapy	Review of cases	Mainly *M. abscessus*, *S. aureus*, *P. aeruginosa*	-	A 70% success rate among 17 patients, with 2 cases of failure.	[124]
TSPphg	Case study	MRSA	Topical application	Significant reduction in bacterial count at 68 µg/mL for 90 min relative to kanamycin.	[125]
AB-PA01	Case report	*P. aeruginosa*	Nebulised and intravenous adjuvant	Successful resolution of the infection, along with the apparent elimination of pathogen colonisation.	[126]
Anti-*K. pneumoniae* phage	Case report	β-Lactamase-positive *K. pneumoniae*	Intravesical and oral routes. Administered with meropenem	Lytic activity of bacteriophage was established using a spot test; however, treatment was specific to a single strain.	[127]
vB_Ts2631	Case study	*Acinetobacter baumannii*, *P. aeruginosa*, and members of Enterobacteriaceae	Endolysins	Electron microscopy of *A. baumannii* showed cell wall damage with cytoplasmic leakage accompanied by apparent signs of cell decay.	[128]
OMKO_1_	Case report	*P. aeruginosa*	Administered as injectables with ceftazidime	No evidence of recurring infection was observed in the patient. Potentially, the phage can attack bacteria.	[129]
ɸABKT21phi3 ɸKpKT21phi1	Case report	*K. pneumoniae*, *A. baumannii*	Intravenous adjuvant with meropenem and colistin	Graft healing and the absence of chronic pain in the patient’s bone were observed. After 8 months, complete absence of pathogens was reported.	[130]
Achromobacter phage cocktail	Case report	MDR *Achromobacter xylosoxidans*	Inhaled and oral routes. Administered with Piperacillin/Tazobactam	Improved lung function indicates the treatment of cystic fibrosis infection in a patient.	[131]
Staphylococcal phage Sb-1	Case report	MRSA	Interstitial/intraosseous and later administered levofloxacin.	Long-term resolution of diabetic foot ulcer with no event of reoccurrence.	[132]

The advancement and integration of phage therapy into mainstream clinical practice are hindered by significant regulatory complexity and standardisation issues, particularly in relation to pharmaceutical regulation, manufacturing practices, and clinical trial design. One of the primary challenges is that standardisation is problematic due to the high specificity of phages to their bacterial hosts, which often necessitates the use of customised or adaptive phage cocktails. This creates difficulties in defining consistent Good Manufacturing Practice (GMP) standards for production. Unlike mass-produced antibiotics, phage preparations may require continuous modification and quality control to match the evolving resistance patterns of target bacteria. Ensuring the purity, potency, and stability of phage preparations across different batches becomes a logistical and scientific challenge, particularly when phage mixtures must be updated rapidly in response to clinical needs [133]. Moreover, standardised clinical trial methodologies for evaluating phage therapy are still underdeveloped. The high degree of biological variability and personalised nature of PT treatment make it difficult to design large-scale, double-blind, placebo-controlled trials, which are the gold standard for pharmaceutical evaluation. This results in regulatory uncertainty about how efficacy and safety should be assessed, especially when comparing results across different studies or treatment settings [109,134,135]. In addition, regulatory inconsistencies between countries further complicate global phage therapy development. For instance, some nations, such as Georgia and Poland, have integrated phage therapy into clinical practice under more permissive frameworks, whereas others adhere strictly to conventional drug approval routes, limiting international collaboration and harmonisation [136]. This lack of unified guidelines impedes the scalability and commercialisation of phage-based products, discouraging investment and slowing innovation.

#### 4.1.3. Microbiome-Based Interventions

The gut microbiota is key in maintaining human health. The microbiota inhibits the activities of infectious pathogens by stimulating the immune response and efficient nutrient utilisation, as well as competing for select resources with other bacteria within certain ecological niches [137]. The gut microbiome is unique to individuals based on genetics and environmental factors, hence the potential of microbiome modulation by introducing exogenous microorganisms to restore balance and eliminate pathogens [138]. Gut microbiome–drug interaction impacts microbiome metabolic activities, resulting in increased bioavailability, increased toxicity, or drug inactivation. Zimmermann et al. [139] investigated the metabolic activity of 76 diverse human gut bacteria on 271 oral drugs. Specifically, over half of the drugs were either partially or completely metabolised by at least one bacterial strain, and each bacterial strain was able to metabolise from 11 to 95 drugs, suggesting a link between gut microbial genes and metabolism, which plays certain roles in individual microbiome variability and drug metabolism. Therapeutic strategies to modulate the gut microbiome mainly involve faecal microbiota transplantation (FMT). However, non-antibiotic prophylactics such as probiotics have also gained widespread popularity [140]. Similarly, genetic engineering has gained a place in the modulation of microbiome-related pathways to enable the production of certain therapeutic molecules in the gut to improve healthcare for microbial disease management.

FMT is a prominent therapeutic approach involving the transfer of faeces from a healthy donor to a patient’s intestine aimed at the decolonisation of the intestinal pathogenic bacteria in the gut environment, recovery of gut diversity, and development of competition to remove pathogens from the residing microbiota [141]. FMT has been proven to be an effective treatment option for recurrent *Clostridioides difficile* infection (RCDI) and may be an effective approach for reducing intestinal antibiotic-resistant bacteria (ARB), as shown in a single-centre study reported by Milan et al. [142]. This study showed that a relatively high diversity of ARB was present in the patient’s gut, which declined upon completion of FMT with a resolution of symptoms and an increased ratio of Bacteroidetes and Firmicutes. Again, Battipaglia et al. [143] investigated FMT before and after allogeneic haematopoietic transplantations in patients with haematologic malignancies carrying MDR carbapenemase-producing bacteria and vancomycin-resistant enterococci. The results showed safe and efficient decolonisation of the gut.

Similarly to FMT, probiotics, prebiotics, and postbiotics are considered important microbiome-based interventions in the treatment of infectious diseases. Prebiotics are considered “food” for the gut microbiota, and probiotics are introduced microbes, while postbiotics, also called inactivated probiotics, are considered the end products of probiotics. These interventions can have high levels of antibacterial activity within the gut. Ibrahim et al. [144] investigated the antimicrobial and antioxidant activity of some probiotics while monitoring the production of vitamins, organic acids, and short-chain fatty acid composition. It was observed that these organisms and their products can effectively remap the microbiome of the gut. Again, in a study [145] that co-cultivated *Enterococcus faecium* with *Ligilactobacillus salivarius* and *Limosilactobacillus reuteri*, it was shown that these organisms produced bacteriocin-like inhibitory substances (BLISs), which had a bacteriostatic effect against selected gut microbiome strains responsible for dysbiosis. Similarly, some postbiotics have also been shown to competitively bind to pathogenic bacteria receptors, change the expression of host genes, and modulate host environmental components [146]. There is growing use of a combination of probiotics and prebiotics in the modulation of the gut microbiome. The International Scientific Association for Probiotics and Prebiotics (ISAPP) defines synbiotics as the combination of live microorganisms (probiotics) alongside substrates (prebiotics) to confer beneficial effects on gut health [147]. The efficiency of symbiotics and common antibiotics in modulating the gut microbiome and general health was analysed using 708 broiler chicks under heat stress (HS) conditions. A higher body weight was observed in chicks fed synbiotics compared to those fed conventional antibiotics. Also, a similar *E. coli* count was observed for both synbiotic- and antibiotic-fed chicks, highlighting their potent antimicrobial potential [148]. Table 3 outlines several microbiome-based interventions that have proven efficient in the control of bacteria, indicating the increasing adoption of probiotics, prebiotics, and synbiotics in the modulation of the gut microbiota. However, in this research area, disregard for ethical and safety concerns and a dearth of clinical trials, with most studies conducted using animal models, are significant obstacles that the field of microbiome treatments must overcome.

Furthermore, while microbiome-based interventions offer promising alternatives to conventional treatments, they also raise significant ethical and safety concerns that must be carefully addressed in clinical and research contexts. One of the foremost ethical considerations is informed consent, which must clearly communicate the biological nature of FMT, including its human stool origin, and the potential risks, benefits, and uncertainties associated with its use. Similarly, given the evolving nature of microbiome science, long-term effects remain largely unknown, making transparency and patient autonomy critical. In addition, the collection and use of donor stool introduce concerns related to privacy and confidentiality. Donors must be assured that their personal health data will be protected and that their biological material will not be used beyond the agreed purpose without further consent. As these therapies extend beyond well-established guidelines, there is the potential ethical issue of their abuse through off-label use, particularly when patients are exposed to unproven interventions without sufficient evidence or regulatory oversight [149]. To mitigate these risks, robust safety protocols are essential. These begin with rigorous donor screening to exclude transmissible pathogens and antimicrobial resistance genes, followed by controlled processing and storage of faecal material under sterile laboratory conditions. Compliance with national and international regulatory frameworks is necessary to ensure that microbiome-based interventions meet established standards for quality and safety [150]. Furthermore, long-term surveillance is crucial to detect delayed effects and to contribute to the broader understanding of microbiome–host interactions. Summarily, while microbiome-based therapies hold transformative potential, their responsible application demands a comprehensive ethical and safety framework that balances innovation with patient protection, public trust, and equitable healthcare delivery.

**Table 3:** Summary of recent microbiome-based interventions and their reported antibacterial activities.

Microbiome-Based Interventions	Type	Bacteria	Antibacterial Activity	Reference
Mulberry and *Bacillus* spp.-derived postbiotics	Postbiotics	*E. faecalis*, *E. coli*, *Salmonella* spp., and *S. aureus*	Variable antibacterial activity against the tested bacterial strains via the formation of complexes with cell walls. MICs of 30–40.5 mg/mL.	[151]
*Ascophillum nodosum* and *Lithothamnium calcareum*	Prebiotics	*E. coli*	Antibacterial activity possibly due to acidity, competition for nutrients, induction of host immune cells, and production of bacteriocins at MICs of 18 and 20 mg/mL.	[94]
*Lacticaseibacillus rhamnosus*	Probiotics	*Streptococcus mutans*	A decrease in the number of viable bacteria indicates antibacterial and antibiofilm potential.	[152]
*Lactobacillus casei* and *L. plantarum*	Postbiotics	*S. mutans*	At MICs of 64 µg/mL and 128 µg/mL, *Lactobacillus casei* and Lactobacillus plantarum inhibited bacteria.	[153]
*Bacillus amyloliquefaciens* and *L. plantarum*	Postbiotics	*E. coli*, *P. aeruginosa*, *Salmonella* spp., *Clostridium* spp., and *S. aureus*	Showed broad-spectrum antimicrobial activity against tested isolates and exhibited an immune response with an MIC of 25 mg/mL.	[154]
*Lactobacillus paracasei* ET-22	Postbiotics	*S. mutans*	The living bacteria, heat-killed bacteria, and secretions of postbiotics showed antibiofilm function.	[155]
*L. plantarum*	Postbiotics	*Salmonella* spp.	The in vitro study showed a triggered AMP-activated protein kinase (AMPK) signalling pathway that induced autophagy with an MIC of 25 mg/mL.	[156]
*L. plantarum* EIR/IF-1	Probiotics	*Prevotella denticola*, *Streptococcus sanguinis*, and *Fusobacterium nucleatum*	Inhibition of microbial growth and inhibited biofilm formation with an MIC of 12.5 mg/mL.	[157]
*Saccharomyces cerevisiae* (PTCC 5269)	Postbiotics	*Salmonella typhi*, *Streptococcus mutans*, *E. coli*, and *Listeria monocytogenes*	Reduced cell viability, suppressed cell division, and induced apoptosis in bacterial cells.	[158]
*Lactobacillus* spp.	Probiotics	*E. faecalis*	A cocktail mix of three species of *Lactobacillus* spp. showed high inhibitory activity relative to a single supernatant and a common treatment option against the bacterium with an MIC of 50 mg/mL.	[159]
*Leuconostoc mesenteroides*	Postbiotics	*Vibrio* spp., *P. aeruginosa*, and *E. coli*	Exerted inhibitive activity on isolates alone and in combination with an essential oil. The leakage of intracellular metabolites and DNA damage was reported at an MIC of 0.5 µg/mL.	[160]
Gut microbiome	FMT	ESBL (*E. coli* and *K. pneumoniae*) *MDR Enterobacter aerogenes*	Change in resistant microbial profile, followed by the absence of resistant species.	[161]
*L. plantarum*	Probiotics	*P. aeruginosa*, *S. typhimurium*, *B.* spp., *E. coli*, and *S. aureus*	Broad-spectrum bacterial inhibitory activity.	[162]
*Phthalyl pullulan* NAPs-treated *L. plantarum*	Synbiotics	*E. coli* and *Listeria monocytogenes*	Antibacterial activity via the production of plantarcin due to intracellular stimulation.	[163]

#### 4.1.4. Metals

Metal ions are being explored as alternatives to antibiotics, either as monotherapy or in combination with other treatment options, such as metallo-antibiotic compounds. The use of metals for therapeutic purposes is an ancient method of treatment. The *Ebers Papyrus* provides the earliest documentation of the use of metals for treatment [164]. Ayurveda represents one of the oldest traditional medicine systems in Asia, with most Ayurvedic products containing metals such as mercury, gold, silver, lead, zinc, copper, etc., in a preparation called Bhasmas. Bhasmas are Ayurvedic metallic herbal concoctions that are made through traditional preparation procedures that include the blending of metals with herbal juices, animal-based products (such as milk, urine, and feathers), and further incineration, which is believed to purify medicines and reduce toxicity [165,166]. It has been reported in a study that investigated components of 252 Ayurvedic products and herbal components that 65% of them contained lead, 38% mercury, and 32% arsenic compounds [167]. Although the toxicity of metal ions in the medicines in this study is in line with recommended doses, they may potentially be hazardous to humans with frequent consumption and can result in possible intoxication and other safety concerns. Other reports assessing the bioaccessibility of lead and arsenic in Ayurvedic medicine indicate that the consumption of the recommended doses of these medicines can increase the bioaccessibility of the metals, which can then exceed the recommended daily intake of toxic elements [168,169]. However, Ayurvedic practitioners argue that Ayurvedic preparation methods in the form of Bhasmas alter the toxicity of their metallic content and hence differ from environmental metal forms [168]. This idea may have lessened the questions about the metal toxicity of Ayurvedic medicine, but questions remain on this.

The therapeutic use of metals in Ayurveda has been extended to the treatment of infectious pathogens of bacterial origin. Due to the problem of dosage toxicity and the possibility of increased metal exposure, nanotechnology has been adopted in the use of metals to enhance their stability and antibacterial activity at extremely low concentrations due to their high surface-to-volume ratio and chemical properties [170]. There are more recent studies in this area. Elmehrath et al. [171] compared the effect of a Cu-1,3,5-benzenetricarboxylate metal–organic framework and copper ions bridged by deprotonated gallate ligands (H^2^gal^2−^) against *E. coli* and *Lactobacillus* spp. The result showed successful inhibition of bacteria by both frameworks due to the internalisation of Cu^2+^ ions and gallic acid by bacteria, which disrupted the cell membrane and halted nucleic acid synthesis. Again, Oetiker et al. [172] analysed the potential of copper oxide (CuO) nanoparticle (NAP) conjugates, and results showed the selective lethality of CuO NAPs against oral commensal species and pathogens. Similar to the mode of action suggested by Elmehrath et al. [171], CuO NAPs disrupt the cellular membrane, altering its permeability, further causing injury to the DNA and obstructing ATP production [172]. Synergistic potential has also been reported with the use of metal against infectious pathogens. Hamid et al. [173] demonstrated the reduction in colony-forming units (CFU) of bacteria isolated from hospital waste pipes when treated with silver nanoparticles (Ag NAPs) and cold plasma separately. However, upon treatment with Ag NAPs and cold plasma simultaneously, a complete antibacterial effect was observed.

Although metallic components by themselves are effective against bacteria, like the antibacterial effect of zinc oxide (ZnO) in the absence of light [174], there is increased antibacterial efficacy of metals as nanoparticles and as nanoparticle–natural product conjugates [175]. For example, when ZnO NAPs are incorporated with plant extracts, there is enhanced antibacterial activity due to the bioactivity of inherent compounds of the extracts, as seen in the case of ZnO NAPs synthesised using *Eucalyptus robusta* [176]. Similarly, the antibacterial effect of Ag NAPs obtained from aloe vera extract was compared to the effects of ciprofloxacin, vancomycin, and ampicillin against *E. coli*, *Pseudomonas luteola*, and *Bacillus subtillis*, with results suggesting that, comparatively, the antibiotics showed relatively weak antibacterial effects [177]. To date, several studies on the antibacterial effect of metals have been reported (Table 4), and progress in this field of research may create a mainstay to combat AMR.

While metals have demonstrated considerable promise as antimicrobial agents, both historically and in modern applications, a balanced appraisal must acknowledge the substantial toxicity risks and long-term safety concerns associated with their use. In traditional systems such as Ayurveda, metal-based preparations like Bhasmas have been used for centuries, yet, as highlighted, studies indicate the presence of toxic elements such as lead, mercury, and arsenic in a significant proportion of these products. Although it is argued that traditional preparation methods reduce toxicity, empirical evidence suggests that chronic use, even at recommended doses, may lead to bioaccumulation and systemic toxicity, particularly when bioaccessibility of metals is enhanced during digestion [193]. These concerns are amplified in vulnerable populations, including pregnant individuals and those with compromised organ function. Moreover, the growing interest in nanotechnology-enhanced metal formulations for antimicrobial use necessitates a critical examination of ecotoxicological impacts, as nanoparticles, due to their high reactivity and small size, may persist in the environment and bioaccumulate in aquatic systems. However, this is a subject for a different discussion, as it is a little-researched area. Overall, there is a need for a more nuanced perspective and a cautious integration of metal-based therapies into clinical practice. One that situates metals as a potentially valuable, but not unproblematic, component of the antimicrobial arsenal. Future research must prioritise risk–benefit analysis, environmental sustainability, and responsible innovation, ensuring that enthusiasm for these agents is matched by rigorous evaluation of their broader implications.

#### 4.1.5. Antimicrobial Peptides (AMPs)

**Figure 2:**
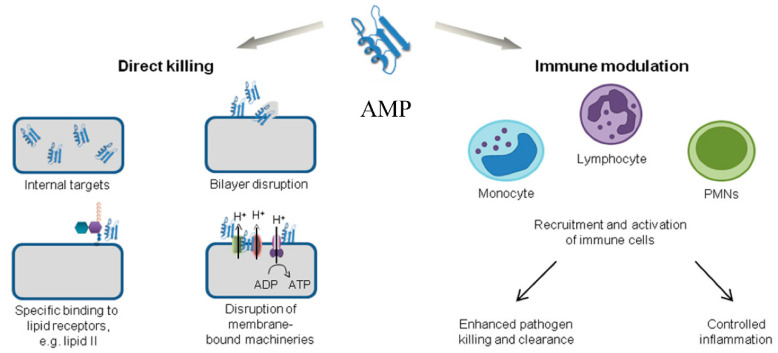
Mechanisms of action and therapeutic targets of AMPs [178].

AMPs are a promising novel class of therapeutic molecules that have shown similar activity to traditional antibiotics [194]. They are small proteins with broad-spectrum antimicrobial activity synthesised mainly by lactic acid bacteria, other bacterial groups, and some plants. Over the past two decades, hundreds of AMPs have been isolated from single-celled organisms, invertebrates, and vertebrates and are being explored for potency against infectious bacteria. AMPs are of interest because they are quick-acting, are naturally synthesised (although synthetic and semi-synthetic variants exist), are easily degraded in the GIT, and do not elicit resistance in bacteria, unlike conventional antibiotics [195,196]. The first AMP (nisin), which is currently utilised as a biopreservative mainly in the food and beverage sector, was isolated from lactic acid bacteria and works by inducing cytotoxicity as a competition strategy against other bacterial species through disruption of bacterial cell walls [197]. The mechanisms of action of AMPs vary but commonly involve pore formation in bacterial membranes, enzymatic degradation of essential cell wall components, or interference with nucleic acid synthesis. These distinct modes of action allow bacteriocins to circumvent some of the resistance mechanisms that compromise conventional antibiotics. Generally, these are categorised into membrane target and non-membrane target mechanisms [198]. While the former operates by penetrating bacterial cells and destroying inherent cellular membranes, thus disrupting cellular function by binding to the bacterial peptidoglycans and perforating cell membranes through linkage with the phospholipids, the latter mechanism is more varied. The mechanisms of action and therapeutic targets of AMPs are visualised in Figure 2.

The biomedical potential of AMPs extends across several domains, including topical applications, gastrointestinal therapies, oral care, and wound management. For instance, nisin, produced by *Lactococcus lactis*, has demonstrated efficacy against multidrug-resistant pathogens such as *Staphylococcus aureus* and *Clostridioides difficile*, and has been incorporated into various formulations for clinical and veterinary use [199,200]. Moreover, AMPs are being explored as adjuncts to antibiotics in combinatory therapies, where they can either enhance the efficacy of existing drugs or restore activity against resistant strains [201]. In biofilm-associated infections, which are some of the most challenging clinical presentations to treat due to their high resistance to antibiotics, AMPs (bacteriocins) have shown potential in disrupting biofilm integrity and enhancing bacterial clearance [202]. Their ability to penetrate biofilms and act on dormant bacterial cells makes them attractive candidates for treating chronic infections involving devices such as catheters, implants, and prosthetics [203].

Furthermore, in the context of antimicrobial resistance control, AMPs offer several strategic benefits. Their narrow spectrum reduces collateral damage to beneficial microbes, thereby preserving host microbiota integrity and reducing the emergence of resistant pathogens [204]. Unlike many antibiotics, resistance to bacteriocins tends to emerge more slowly, reducing the likelihood of widespread resistance dissemination. However, challenges remain in translating AMPs into mainstream clinical use. These include issues of stability, scalability of production, immunogenicity, and delivery in systemic infections [205]. Advances in synthetic biology and peptide engineering are addressing some of these limitations by enabling the design of more stable and potent AMP derivatives with extended half-lives and improved pharmacokinetics. Ultimately, AMPs represent a valuable addition to the antimicrobial arsenal, particularly in this era of rising drug resistance. Presently, the AMP database [Data Repository of Antimicrobial Peptides (DRAMP), http://dramp.cpu-bioinfor.org/, accessed on 1st May 2025] contains 29,948 entries for AMPs, some of which are clinically relevant against bacteria [206]. This is outlined in Table 5.

While not currently positioned as a complete replacement for antibiotics due to the limited number of AMP candidates that make it to and through clinical trials, their integration into targeted therapies, combination regimens, and preventive healthcare strategies has the potential to reshape the management of infectious diseases. Recently, several advances and technologies have been developed to harness the potency of AMPs and broaden clinical applications, such as the artificial synthesis of multifunctional peptides, cell-penetrating peptides, and peptide–drug conjugates [225]. This has brought about progress in this research area, although proteolytic and gastric degradation of AMPs in the stomach and GIT still pose a challenge for oral delivery. To combat this, novel delivery methods have emerged to improve AMPs’ stability. Continued investment in research, regulatory clarity, and clinical trials will be essential to fully realise the biomedical promise of bacteriocins in controlling antimicrobial drug resistance and to solve some of these underlying scientific challenges.

#### 4.1.6. Immunomodulating Agents

Immunomodulators are substances that modify the activity of the immune system. They can be immunostimulants, which stimulate the immune system, or immunosuppressants that suppress it. Early use of immunomodulating agents can be traced to the use of serum therapy and antibody preparation in the 1920s, even before the advent of antibiotics and “the golden age” of antibiotics, which limited their usage. Current immunomodulating agents are mainly targeted monoclonal antibodies (mAbs), which have seen a growing uptake, especially in the treatment of cancers, autoimmune diseases, and some infections. In recent years, developments have been made to improve the performance of monoclonal antibodies (mAbs) as therapeutics, and these have been used synergistically for the control of infectious diseases [226]. Several reports have been made around the use of immunotherapy in recent times; however, some immunomodulators have shown inconsistent results in clinical trials.

The use of immunomodulators is based on the regulation of the innate and adaptive immune responses of the host and depends on two approaches: antigen-specific immunomodulators, which include antibody therapies and the use of vaccines, and non-antigen-specific immunomodulators, such as the use of AMPs (Table 5) and microbiome-based therapies [227], which have been described previously. Antibody therapies rely on the use of monoclonal antibodies (MAbs) to target and bind virulent factors of pathogens, such as binding to invading bacterial toxins to form antibody–toxin complexes, which are subsequently cleared by the reticuloendothelial system [228]. MAbs have also been observed to exert bactericidal effects by inducing phagocytosis through activation of the antibody-dependent cellular cytotoxicity (ADCC) pathway and activation of the complement system, leading to the elimination of pathogens by the complement-dependent cytotoxicity (CDC) pathway [229]. The mode of action of MAbs reduces the likelihood of resistance development and may be associated with a low risk of resistant mutants’ selection, as shown by the study by Tkaczyk et al. [230], in which mutations in the *S. aureus* alpha-toxin epitope targeted by MAb MEDI4893 reduced bacterial fitness upon infection.

Another area of interest in immunotherapy is the use of vaccine-based immunotherapeutic agents, which have been used for decades and have a low probability of AMR resistance development [231]. Presently, the use of vaccines to curb AMR is gaining attention, with 94 new vaccine candidates currently under active preclinical development and 61 in the active clinical development stage [232]. Research in prophylactic and therapeutic vaccines is expanding, with reverse vaccinology, novel adjuvants, bioconjugates, and rationally designed bacterial outer membrane vesicles (OMVs) being new technologies and approaches currently being employed in advancing research on the use of vaccines as antibacterial agents [233]. Vaccines have the potential to be mainstream cost-effective treatment options against MDR, particularly in middle-income and low-income countries with high disease burdens [234].

#### 4.1.7. Antimicrobial Enzymes

Enzymes are major components of living things and are used in the catalysis of biological and biochemical reactions [235]. Enzymes are commonly utilised to accelerate bioprocesses, but were employed before the antibiotic era in wound healing and other treatments, such as tooth extraction [236]. However, the use of enzymes decreased upon the discovery of their non-specificity in the 1900s. In 1922, the development of insulin revolutionised the exploration of enzyme-linked therapeutics, followed by advancements in protein purification techniques. In the 1950s, the potential of enzymes in wound treatment and treatment of bacterial infections was explored in detail [237]. However, there were limitations to the use of enzymes therapeutically due to studies indicating that they induce haemorrhage and lethality in rats, which reduced their applications. In recent times, newer research has explored the use of enzymes as alternatives to antibiotic therapies, with several reports showing the efficacy and potential of enzymes in curbing bacterial infection (Table 6).

Enzymes can be effective in the disruption of bacterial biofilms. Polybacterial infection allows the formation of biofilms that enhance AMR by the secretion of extracellular polymeric substance (EPS), which protects bacteria from antibiotics, creating a high tolerance level against them [254]. Current studies have focused on the use of enzymes as monotherapy and synergistically with traditional antibiotics in the elimination of bacterial biofilm to prevent resistance development in bacterial populations while inhibiting growth. Lysozyme obtained from chicken has been reported to attack the cell wall of bacteria and hence is used in antibacterial wound coatings [255]. Leonarta and Lee [256] adopted the use of a polyvinyl alcohol (PVA) membrane made of encapsulated glucose oxidase (GOx) and glucose (Glu) nanofibres to produce hydrogen peroxide (H_2_O_2_) in the presence of an aqueous solution, which showed activity against *E. coli* and *S. aureus*. Similarly, Lyagin et al. [257] compared the efficiency of two coatings made of quorum-quenching enzymes with antibiotics (polymyxins), metal NAPs, and enzymes on fibre materials. This study reported diverse antibacterial activity among groups with variable specificity; for example, tantalum NAPs acted preferentially against *E. coli*. Another approach for the use of enzymes in the control of bacterial infections relies on the eradication of EPS to inhibit biofilm formation and bacterial growth [254]. The use of trypsin, β-glucosidase, and DNase I, which target proteins, polysaccharides, and extracellular DNA (eDNA), is effective in bacterial and biofilm control, as these are the main components of bacterial EPS [254]. In a recent study, treatment with trypsin/DNase I resulted in the dispersion and dissolution of *S. aureus–P. aeruginosa* biofilms [254]. Several other studies have reported similar trends, including the obstruction of N-acyl homoserine lactones (AHLs) by lactonases that work by disrupting the lactose ring in AHLs [258], amidases that work by hydrolysis of AHLs’ amide bonds, and oxidoreductases that cause oxidation of the acyl chain of AHLs [259]. Additionally, several quorum-quenching enzymes have been reported to limit the activity of virulence factors, motility, and biofilm formation in bacteria [259,260]. Most enzymes show no toxicity and little to no effects on the host, thus promising to be good therapeutic candidates. However, certain limitations to their use have been identified due to instability, degradation by host and bacterial proteases, and pharmacokinetics.

## 5. Roles of Antibiotic Alternatives in Mitigation of AMR

As a chunk of existing antibiotics are becoming ineffective due to drug-resistant microbes and proliferation of AMR genes, natural alternatives (NAs) to the antibiotics appear to be an innovative approach to addressing this problem. As has been described, some NAs work by silencing resistance, disrupting bacterial processes, and improving host defence and synergistic defence mechanisms, amongst others. Some of these alternatives have seen steady usage, e.g., bacteriophages, and others are in various stages of development. The probability of antimicrobial resistance (AMR) developing against NAs is generally lower than that of traditional antibiotics due to their complex structures, multi-targeted mechanisms of action, and lower clinical usage. Many NAs, such as plant extracts and antimicrobial peptides, exert their effects on multiple cellular targets, reducing the likelihood of resistance development. However, resistance can still occur, particularly with repeated exposure or suboptimal dosing, as seen with some peptides and metal-based agents. Therefore, while NAs offer a promising alternative, prudent use and ongoing surveillance are essential.

A major role of NAs in combating AMR is in the development of potent drug candidates that could act against bacterial pathogens that adopt a distinct mechanism of action away from traditional antibiotics to evade AMR development while inhibiting bacterial pathogens [261]. Even when the exact mechanism of action of an NA is not fully understood, it can still be used logically and effectively, relying on empirical evidence, traditional knowledge, in vitro and in vivo testing, observed clinical outcomes, and modern analytical tools that support its safety and therapeutic potential. Additionally, NAs may act as supplementary therapeutics in a combinatorial approach to reanimate antibiotics and neutralise bacterial defences. For example, Rajamanickam et al. [262] reported improved efficacy of the antibiotic ceftiofur in combination with the phytochemical phosphorylcholine against *Staphylococcus epidermidis* and *S. aureus.*

Overall, NAs represent a sustainable source of new antibacterial drug candidates in the drug development pipeline due to their abundance in nature and non-toxic environmental impact and spectrum of activity. For example, plants are highly biodiverse, with about 374,000 identified species, of which there are approximately 290,000 unstudied plant species with possible antibacterial potential [263]. Additionally, relative to synthetic drug options with mild to intense side effects, NAs such as plants are indispensable due to related human safety, non-toxicity, and ease of degradation [264]. The development of a synergistic effect between new drug candidates and existing drugs and antibiotics has proven to be efficient in reinstating bacterial vulnerability to treatment and serves as a direct solution to the problem of MDR bacteria. For example, Fujiki et al. [265] demonstrated that phages show the potential to reverse antibiotic resistance. The indiscriminate use of antibiotics on farm animals as prophylaxis and growth promoters is an active source for the spread of resistant bacteria and AMR genes into the environment via food products, waste, and direct contact, indirectly making zoonotic pathogens reservoirs of antimicrobial resistance genes to human pathogens [266,267]. Addressing AMR spread via livestock farming will require a multifaceted approach involving the use of NAs such as vaccines to control disease incidence while protecting animals’ welfare [268]. The development of NAs and their utilisation as therapeutic agents will require further research to overcome present challenges. However, they may not necessarily replace traditional antibiotics but could work to increase their efficacy and alter the traditional pattern of resistance development in bacteria.

## 6. Current Challenges in Implementation

It is believed that certain drugs can be effective but not without side effects, and the level of side effects can make the drug unsafe for consumption. The perception that NPs and NAs are inherently safe and pure may not always be true, and certain concerns about the safety of NAs have sprung up; for example, medicinal plants and their derivatives have been reported to be effective against certain infectious pathogens, but a complex interaction exists between plants and their environment, which makes it difficult to generalise the effect of abiotic stress on plant species, which may affect the efficacy of their biological compounds [269,270]. Additionally, data regarding the use of NAs in the treatment of infectious diseases in humans are not conclusive and sometimes present conflicting or negative results, which are further complicated by challenges in trial design such as non-randomised and non-placebo-controlled studies [271,272]. There are other concerns with regard to the applicability of these NAs therapeutically. For example, metals have been found to have significant toxic effects on various cultured mammalian cells. This is highlighted in a recent study where AgNPs were toxic to immune cells, including mononuclear cells, where they exhibited both time- and dose-dependent toxicity [273]. Similarly, in *Drosophila melanogaster*, oxidative stress and upregulation of heat shock protein (HSP 70) expression were induced by AgNPs at doses of 50–100 μg/mL for 24–48 h [274]. There is also the challenge of therapeutic dosage, as the pharmacokinetics of AMPs highlight the potential concern of a short half-life and rapid elimination from systemic circulation, which may affect the bioavailability of these compounds for effective elimination/clearance of infections, thus limiting their clinical applicability [275]. Additionally, certain AMPs classified as receptor-binding peptides exert antibacterial effects via immune modulation. This group of AMPs has the potential for off-target effects, causing additional chronic inflammatory diseases, raising concerns about cytotoxicity and poor selectivity [276]. Similarly, bacteriophages carry a potential risk of endotoxin contamination, which may trigger an inflammatory cytokine response and lead to severe health complications, including toxic shock [277].

Technical and logistical challenges in NA research, such as financial constraints, poor resource support, and the lack of technical expertise to develop novel drugs, challenge their development and implementation [25]. Microbial exposure, resistance, and stability of interventions may also pose an issue in the use of NAs, which may limit the clinical implementation of their use. For example, several reports on the failure of staphylococcal vaccines in humans but success in mice have been linked to prior exposure to *S. aureus* by the human immune system, thus causing a reprogramming and rendering the vaccine inefficient [278,279]. Interestingly, daptomycin–methicillin-resistant *S. aureus* has emerged from the use of daptomycin, a peptide-based antibiotic, and mutant isolates have been found to be resistant to cationic AMPs and cross-resistant to vancomycin, which has a different mechanism of action mediated by a multiple peptide resistance factor (MprF) [280,281]. Although this resistance is still considered low in the treatment of staphylococci infections, and newly developed random AMP cocktails are considered efficient substitutes for the treatment of *Staphylococcus*, this development raises some concern [282,283]. It has been suggested that certain bacteria can evolve rapidly and become resistant to AMPs in vitro [284]. Thus, there are questions around the sustained efficiency of these alternatives in the face of bacterial evolution and the inefficiency it imposes on other treatment options in vivo.

## 7. Recent Trends in Drug Discovery, Future Directions, and Research Needs

Due to the challenging nature of finding new antimicrobial compounds, it is relevant to understand the exact molecular targets and possible pathways to facilitate optimisation and dampen off-target effects [285]. Emerging technologies in bioinformatics, genomics, and proteomics create computational and analytic information on the whole genome of a species, which provides new insights into novel drug targets and an understanding of drug mechanisms of action. Several high-throughput screening (HTS) and computational modelling technologies expedite the identification and analysis of vast amounts of potential bioactive compounds and overcome present challenges of natural product library (NPL) screening [286]. Recently, Yüce and Morlock [287] outlined a high-throughput high-performance thin-layer chromatography-based antibacterial assay that enabled the synthesis, identification, and parallel screening of products from organic reactions at a miniaturised scale. Their methods identified 10 antibacterial agents against *B. subtilis* and *Aliivibrio fischeri* with high activity relative to the antibiotic reference ciprofloxacin. Similarly, Wang et al. [288] developed an HTS assay that screened 1261 products and identified 4 NPs that did not directly exert an antibacterial effect but significantly augmented the antibacterial activity.

Other cutting-edge technologies, such as 3D bioprinting and related advanced imaging methods, have been explored for preclinical testing of numerous drugs [289]. Similarly, organ-on-a-chip (OOAC) systems have gained more prominence over the years for the prediction of a drug’s clinical applications as they supersede the use of animal models due to the problem of interspecies differences [210,290,291]. OOAC has evolved to the current use of multi-organ-on-a-chip (multi-OOAC), which comprises an array of organoids or tissues in a single microfluidic device to mimic the single function of an organ, as well as intercommunication between organs [210]. Since many studies have linked gut dysbiosis to the onset of diseases, the pathogenesis of symbiotic interactions and pathogen-induced infectious diseases has been vastly studied using this technology. Lu et al. [292] worked on *Lactobacillus acidophilus* and observed that the bacteria inhibited *Salmonella* invasion in a colon organoid, indicating its potential use as a probiotic, as it showed an increased expression of a major gel-forming mucin that reduced the damage caused by the pathogen. Similarly, Wu et al. [293] used intestinal organoids to demonstrate that *Lactobacillus reuteri* maintained the intestinal mucosal barrier integrity by activating the Wnt/β-catenin pathway and stimulating the repair of epithelial damage by intestinal epithelial proliferation. Using a different experimental approach, Koshovyi et al. [294] used 3D bioprinting for the development of a semi-solid extrusion product from *Eucalyptus viminalis* L. with significant anti-staphylococcal activity as an immediate-release oral delivery system. In line with these advances, several NAs have emerged as strong candidates for clinical translation. Notably, *Teixobactin*, a novel antibiotic isolated from *Eleftheria terrae*, a soil bacterium, has demonstrated potent activity against Gram-positive pathogens, including *Staphylococcus aureus* and *Mycobacterium tuberculosis*, with no detectable resistance in vitro, making it one of the most promising antibiotic discoveries in recent years [295]. *Phage therapy* has also reemerged as a viable treatment strategy for MDR infections, especially with personalised phage cocktails currently undergoing clinical trials and compassionate-use protocols in the US and Europe [296,297]. Additionally, synthetic antimicrobial peptides like *Omiganan* (a cationic peptide derived from indolicidin) are being evaluated in clinical formulations for topical use against *Staphylococcus aureus* and other skin pathogens due to their broad-spectrum activity and low resistance development [298]. These examples clearly show how natural alternatives are gaining ground toward real-world therapeutic use, supported by advances in screening technologies and preclinical modelling. Also, with regard to drug development and repurposing, artificial intelligence (AI), especially machine and deep learning (ML/DL) technologies, is being employed more actively in the testing and repurposing of drugs and in trial analytics within the pharmaceutical industry [299]. Also, AI can play more involved roles in the prediction of novel antibacterial compounds [300], potential resistance sites [301], and other drug dynamics [302]. In addition to tracking the long-term safety of drugs, AI may be adopted in regulatory informatics, real-world data analytics, and pharmacovigilance to support regulatory decision-making and post-market surveillance.

Interdisciplinary approaches and collaborations in drug development are paramount in achieving the global aim of eradicating the problem of AMR. The research and academic sector is highly invested in the development and validation of drug candidates for preclinical trials. However, research is currently limited by a range of factors, especially the lack of funding [303]. Partnerships with external funders such as the pharmaceutical industry will spearhead relevant innovation via research-driven funding and infrastructure contributions. Stakeholders such as the Global Antibiotic Research and Development Partnership (GARDP), Combating Antibiotic-Resistant Bacteria Biopharmaceutical Accelerator (CARB-X), Innovative Medicines Initiative (IMI), and other stakeholders are strengthening public and private partnerships for antibacterial-related research by providing research funding to accelerate the development and global availability of new treatment options [303,304,305]. Although research funding and infrastructure may not be sufficient to engage researchers in the development of drugs outside the pharmaceutical sector, it may be necessary to incentivise research for the development of new antibacterial agents by independent academic research teams. Anderson et al. [25] suggest that the problem of declining profitability and financial losses accrued by antibacterial agent developers has reduced interest in antibacterial innovation and may be resolved by the introduction of “subscription payments, market entry rewards, transferable exclusivity extensions, and milestone payments” as incentives, with subscription payments replacing sales incentives in drug distribution. Furthermore, from the regulatory perspective, differences in pharmaceutical regulation across jurisdictions in approving novel drugs impact global drug approval and cost. For example, the United States and the European Union approach drug regulations differently [306]; hence, there is a need for collaboration to create and harmonise standards across countries to aid in drug development.

Despite the promising advantages of NAs, most therapies are poorly explored due to non-standardised clinical approaches and safety thresholds that undermine confidence in their application. Furthermore, some approved alternatives to antibiotics have reportedly lost potency against some bacteria. For example, evidence suggests that *S. aureus* employs adaptive mechanisms to increase cell membrane stability and circumvent killing by AMPs [307]. Similarly, the toxicity of some alternatives has raised concerns in clinical applications. Although the small sizes of NAPs help them to exert a substantial antibacterial activity, these compounds have high cytotoxicity, limiting their use as their short- and long-term effects remain unknown [308,309]. Similarly, questions about the pharmacokinetics and pharmacodynamics of some of these alternatives remain unclear. Side effects from the usage of alternative therapy have sometimes resulted in death. For example, two cases of norovirus contamination were reported after faecal transplantation, and other cases of bacterial infection resulting in death have been reported [308]. Further questions are as follows: Is the use of these drugs patient-centric? Are they successful only in a small group of people, or do they function as a treatment option for many people? These gaps indicate that the future of new therapeutics lies in constant evaluation, novelty, and collaboration among stakeholders to foster ongoing research in their development.

To guide future research and policy investment, a prioritisation framework is needed and should consider three dimensions: clinical efficacy against resistant pathogens, safety and pharmacokinetic profile, and feasibility of large-scale production and regulation. Through this lens, bacteriophages and AMPs merit immediate translational focus, supported by targeted clinical trials and innovation in delivery technologies. AMPs and metals also should be prioritised for formulation improvements and toxicity reduction, while microbiome-based approaches require long-term cohort studies and clearer regulatory pathways. Importantly, outside the laboratory and clinical settings, economic constraints, including the cost of production, lack of commercial incentives, and limited market viability in low-resource settings, remain substantial barriers to widespread adoption. This is complicated by regulatory hurdles, particularly the absence of harmonised international frameworks for biologics and personalised therapeutics like phage therapy, which further delay integration into mainstream healthcare. Overcoming these challenges will require coordinated efforts across research, policy, and public health domains. This can be achieved by strengthening antimicrobial stewardship programmes, investing in scalable production platforms, and developing adaptive regulatory models. This will offer a realistic path to mainstreaming the use of natural products in mitigating the AMR crisis and safeguarding the future of infectious disease management.

## 8. Conclusions

The development of alternative antibacterial agents is imperative in addressing the escalating global threat of antimicrobial resistance (AMR), which poses profound challenges to public health systems and economic stability worldwide. This review has explored a range of promising alternatives, including plant-derived compounds, bacteriophages, metals, antimicrobial peptides, enzymes, immunomodulating agents, and microbiome-based interventions, for mitigating the emergence and spread of antibiotic-resistant bacteria, highlighting their increasing therapeutic potential. These agents have demonstrated efficacy in both monotherapy and synergistic combinations with conventional antibiotics, offering new avenues for prophylaxis, therapeutic intervention, growth promotion, and immune modulation with varying degrees of efficacy, mechanism diversity, and translational readiness. Despite these advances, significant challenges remain. Among these, bacteriophages and AMPs emerge as particularly promising due to their specificity, ability to disrupt biofilms, and lower risk of inducing widespread resistance. However, concerns persist regarding the incomplete understanding of the mechanisms of action of many of these alternatives, as well as their pharmacokinetics, toxicity profiles, and long-term safety. For example, while metals and antimicrobial peptides offer potent antimicrobial activity, issues such as bioaccumulation, proteolytic degradation, delivery limitations, and production costs hinder their clinical translation. Similarly, microbiome-based therapies, although promising, raise ethical and regulatory questions around donor safety, infection risk, and treatment standards. In addition, despite encouraging in vitro and preclinical data, most natural alternatives remain at the exploratory or experimental stages, and few have advanced to late-phase clinical trials or regulatory approval. Addressing the threat of AMR using natural products will require more than just exploratory research into novel therapeutics; it necessitates a holistic and integrated approach. Strengthening global antimicrobial stewardship programmes, particularly in low- and middle-income countries, enhancing surveillance systems, and investing in basic and translational research geared towards natural alternatives to antibiotics are essential components of this effort. Furthermore, a deeper understanding of bacterial resistance mechanisms and host–pathogen interactions will be crucial in guiding the rational design and clinical deployment of these natural alternatives. Ultimately, advancing these therapies from experimental to clinical application, while ensuring safety, accessibility, and efficacy, will enable researchers and clinicians to provide more targeted, sustainable, and patient-centred antimicrobial care, thereby contributing meaningfully to the global effort to curb antimicrobial drug resistance.

## Figures and Tables

**Table 1:** Recent reports on the use of plant phytochemical compounds as antibacterial agents.

Plant	Phytochemical Compound	Bacterial Isolates	Test MIC (mg/mL)	Positive Control MIC (mg/mL)	Reference
*Withania somnifera* (L.) (Ethanolic extract)	Alkaloids, tannins, steroids, and flavonoids	*S. aureus*	2	0.625	[86]
		*E. coli*	2	0.352	
*Calpurina aurea* (Ethanolic extract)	-	*S. aureus*, *Pseudomonas aeruginosa*, *Salmonella typhi*, *E. coli*, and *Shigella dysenteriae*	1.56	0	[90]
*Phoenix dactylifera* L. (Hydro-ethanolic extract)	Flavonoids and phenols	*S. typhi*, *H. pylori*, *E. coli*, and *S. aureus*	22	0	[91]
*Allium cepa* (Ethanolic extract)	Phenol and flavonoids	*S. aureus*, *Enterobacter faecalis*, *E. coli.*, *Proteus mirabilis*, *P. aeruginosa*, *Klebsiella aerogenes*, *Salmonella enterica*, and *Shigella sonnei*	0.78–12.50	0.008–2.50	[92]
*Phoenix dactylifera* (Methanolic extract)	Ketones, lipids, phenols, terpenes, steroids, vitamins, acids, alcohol, etc.	*Staphylococcus* spp., *P. aeruginosa*, *E. faecalis*, *K. pneumoniae*, and *Enterobacter cloacae*	0.7–1.4	0.312–2.5	[93]
*Lithothamnium calcareum*, *Ascophillum nodosum*	Polyphenol, fatty acid, alcohol, and flavonoids	*E. coli*	13	-	[94]
*Carica papaya*	Flavonoids, alkaloids, and saponin	*E. coli*, *S. aureus*, and *P. aeruginosa*	-	-	[95]
*Allium sativum* (Garlic) *Zingiber officinale* (Ginger)	Allicin Essential oil and phenols	*E. coli* and *Salmonella* spp.	0.312 and 0.625	-	[96]
*Allium sativum* (n-hexane fraction)	Alkaloids, phenolics, saponins, flavonoids, coumarin, and triterpenoids	*P. aeruginosa*, *Salmonella typhi*, *E. coli*, *S. aureus*, *Bacillus subtills*, and *S. pneumoniae*	1.562–12.5	7.810	[97]
*Pulicaria crispa* *Pulicaria undulata* (essential oil extract)	Saponins, coumarin, tannins, steroids, and triterpenoids	*S. aureus*, *B. subtills*, *P. aeruginosa*, and *E. coli*	6.25–25	-	[98]
*Tephrosia bracteolate* (Ethyl acetate extract)	Alkaloids, tannins, steroids, and flavonoids	*S. aureus* and *B. subtills*	6.25	10	[99]

**Table 4:** Advances in the application of metals and their complexes as antibacterial agents.

Metal	Metal Complex	Bacteria	Result	Reference
Copper (Cu)	Cu-1,2,3-benzenetricarboxylate	*E. coli* and *Lactobacillus* spp.	Bacterial cell membrane disruption.	[171]
Zinc (Zn)	Zinc sulfate monohydrate (ZnSO_4_.H_2_O)	*E. coli* and *Enterococcus* spp.	Significant decreases in MIC values for *E. coli* with ciprofloxacin and nalidixic acid; however, no significant change was observed with *Enterococcus* spp.	[178]
Cobalt (Co) and Nickel (Ni)	CoNAPS and NiNAPS	*V. cholerae*, MDR *E. coli*, *S. aureus*, and *S. enterica*	Both NAPs showed antibacterial activity against all tested bacteria. NiNAPs showed a relatively higher activity at a low concentration.	[179]
Zinc (Zn)	Zn (II) sulfate monohydrate (ZnSO_4_.H_2_O), Zn (II) sulfate heptahydrate (ZnSO_4_.6H_2_O), Zn (II) chelate of protein hydrolysate, Zn (II)chelate of amino acid hydrate, and Zn (II) chelate of glycine hydrate	*E. coli*, *S. aureus*, *P. aeruginosa*, and *Klebsiella oxytoca*	Findings showed specificity and dose dependency in the action of Zn (II) sulfates and Zn (II) amino acid complexes; however, disruption of biofilm formation was also observed. Antibacterial activity was observed with all complexes.	[180]
Titanium (Ti)	Titanium dioxide (TiO_2_) and Titanium silver (TiAg)	*E. coli* and *S. aureus*	Antibacterial rates of 99.91% and 97.38% were observed for *S. aureus* on the first and tenth days, respectively. The observed values were noted to be 99.02% and 95.13% for *E. coli.*	[181]
Silver (Ag)	Starch-based polyurethane nanocomposites (magnetic NAPs and silver NAPs)	*E. coli* and *S. aureus*	The nanocomposite showed high antibacterial activity, which increased with an increase in concentration.	[182]
Silver (Ag)	Silver nanoparticles (AgNAPs)	*S. aureus*	A decrease in membrane integrity of 45.8% in the isolate and reduced membrane potential based on flow cytometry. In vivo, the analysis showed healing of wounds.	[183]
Calcium (Ca)	Calcium oxide (Ca) NAPs	*K. pneumoniae* and *S. aureus*	The different laser energies showed variations in antibacterial activity.	[184]
Aluminium	Aluminium oxide (AlO_3_) NAPs	*P. vulgaris* and *Streptococcus pyogenes*	Antibacterial activity was observed at 200 µg/mL with high lethality on *P. vulgaris.*	[185]
Iron (Fe)	Iron oxide (Fe_3_O_4_)–copper metal–organic framework (Cu-MOF)	Gram-positive and Gram-negative	Stabilises the MOF to enable persistent Cu release that kills bacteria; however, it does not show significant antibacterial activity.	[186]
Magnesium	Magnesium oxide (MgO) NAPs	*E. faecalis*, *E. coli*, *S. aureus*, and *Shigella dysenteriae*	Antibacterial activity against all tested bacteria with MICs of 300–550 µg/ml.	[187]
Gold (Au)	Ciprofloxacin-loaded gold NAPs (CIP-AuNAPs)	*E. faecalis*	In vitro, assessment of metal complex and ciprofloxacin showed MICs of 1 µg/mL and 2 µg/mL, with a lower bacterial load observed in mice treated with CIP-AuNAPs.	[188]
Titanium (Ti)	Titanium oxide (TiO_2_) NAPs	*E. coli* and *S. aureus*	Cellular disruption of cells by cell membrane penetration. Induces an abundance of oxidative stress proteins, which impairs biofilm formation.	[189]
Cobalt (Co)	Cobalt (II) sulfate heptahydrate (CoCl_2_.6H_2_O) NAPs	*E. coli*, *S. aureus*, and *P. vulgaris*	Variable-defined clear ZOI was observed for all tested bacteria, with the highest antibacterial activity at 100 µg/mL.	[190]
Cobalt (Co) and Nickel (Ni)	Cobalt (II) sulfate heptahydrate (CoCl_2_.6H_2_O) + ammonium chloride (NH_4_Cl) and Nikel (II) sulfate heptahydrate + hydrogen peroxide (H_2_O_2_) + Hydrochloric acid (HCL)	*S. aureus*	Antibacterial activity was higher with cobalt complexes. The activity based on ZOI was higher on nutrient agar with higher concentration; however, antibacterial activity was relatively lower on blood agar regardless of the concentration.	[191]
Copper (Cu)	Copper (II) sulfate pentahydrate (CuSO_4_.6H_2_O)	*Enterobacteriaceae*, *Staphylococci*, and *Pseudomonas*	Potent against 52% tested isolates with MICs between 100 and 200 µg/mL, with the greatest bactericidal effect shown at an MBC of 1600 µg/mL.	[192]

**Table 5:** Antibacterial activity and modes of action of antimicrobial peptides (AMPs) selected from recent studies based on diverse biological sources, broad-spectrum efficacy, and mechanistic insights.

Peptide	Source	Bacteria	Mode of Action	Reference
Chlorin-e6	*Spirulina maxima*	Multi-species biofilm	Significantly impacted bacterial viability.	[207]
K11	Cecropin A1, melittin, and magainin 2	*K. pneumoniae*	Increased potency of combined antibiotics and prevented biofilm formation.	[208]
EWAMP-R	*Eisenia andrei*	*S. aureus* and *E. coli*	The peptide binds to phospholipids of *S. aureus* and inserts into *E. coli* membrane triggering apoptosis.	[209]
MOp3	*Moringa oleifera*	*S. aureus*	Interacts with DNA gyrase and dihydrofolate reductase to damage the cell membrane.	[210]
SpPR-AMP1	*Scylla paramamosain*	*Vibrio campbelli*	Membrane disruption in bacterial cells and host immune system modulation in vivo.	[211]
Jelleine-Ic	*Apis mellifera*	*Pseudomonas syringae*	Destroyed the cell membrane, induced intracellular reactive oxygen, reduced esterase activity, and altered DNA replication.	[212]
Hylin a1	*Hypsiboas albopunctayus*	MDR *A. baumannii*	Disruption of the bacterial membrane and its permeability by binding to lipopolysaccharide.	[213]
gcIFN-20	Ctenopharyngodon idella	*Streptococcus* spp., *K. pneumoniae*, *P. aeruginosa*, *S. aureus*, and *E. coli*	Interacts with bacterial lipopolysaccharide and causes aggregation and neutralisation. Disrupts the cell membrane and inhibits protein synthesis.	[214]
TroNKL-27	*Trachinotus ovatus*	*Edwardsiella tarda*, *Streptococcus agalactiae*, *Vibrio* spp., *S. aureus*, and *E. coli*	Degrade bacterial genomic DNA and alter cell integrity, causing leakage of cellular contents.	[215]
KR-12-3	LL-37 derivative	*Streptococcus gordonii*	Disrupts the cell wall, reduces the production of inflammatory cytokines, and deregulates bacterial genes responsible for adhesion.	[216]
Pt5-1c	Phosvitin	*K. pneumoniae*, *S. aureus*, and *E. coli*	Restored the sensitivity of bacteria to the tested antibiotics.	[217]
NKL-24	Danio rerio NK-lysin	*Vibrio parahaemolyticus*	Exhibits active membrane cell-killing, reduces bacterial movement, and downregulates transcription genes associated with bacterial virulence.	[218]
Kassinatuerin-3	*Kassina senegalensis*	MRSA, *P. aeruginosa*, *E. faecalis*, *S. aureus*, and *E. coli*	Inactive against Gram-negative bacteria. Disruption of the cell membrane of Gram-positive bacteria.	[219]
OM19R	Oncocin and MDAP-2	*E. coli*, *K. pneumoniae*, *Salmonella* spp., and *Shigella* spp.	Selectively inhibit bacterial cells without initiating cell lysis.	[220]
OVTp12	Ovotransferrin	*S. aureus* and *E. coli*	Disruption of cell integrity and a significant increase in membrane permeability.	[221]
P5	Cecropin A-magainin 2 hybrid	*P. aeruginosa*, *B. subtills*, *A. baumannii*, and *S. aureus*	Destruction of inner and outer bacterial membranes and increased membrane permeability.	[222]
CF-14	Catfish	*Shewanella putrefaciens*	Cell wall penetration and accumulation in bacterial cells, with slight toxicity in red blood cells.	[223]
KC246043.1	*Bacillus megaterium*	*Micrococcus luteus*, *Salmonella typhi*, *P. aeruginosa*, *S. aureus*, and *E. coli*	Broad-spectrum antibacterial activity.	[224]

**Table 6:** Antibacterial activities of common enzymes.

Enzymes	Source	Bacteria Isolates	Mode of Action	Reference
Keratinase	*Norcardia* sp	*Streptococcus mutans*, *S. typhi*, and *K. pneumoniae*	Hydrolyses proteins on the bacterial surface	[238]
Protease SH21	*Bacillus siamensis*	*E. coli*, *S. aureus*, and *Micrococcus luteus*	Disrupts the cell membrane	[239]
Hydrolase Lys14579	*Bacillus cereus*	*B. cereus*	Induced cell wall degradation	[240]
β-1,3-1,4-Glucanase	*Halomonas meridiana* ES021	*B. subtillis*, *Streptococcus agalactiae*, and *Vibrio damsela*	Isolate-dependent antibacterial effect	[241]
Lysozyme	Hen egg white	*E. coli*, *P. aeruginosa*, and *B. subtilis*	Lyses the bacterial cell wall	[242]
Amylase	*Streptococcus pyogenes*	*Streptococcus salivarius*	Inhibits biofilm formation	[243]
Keratinase	*A. baumannii*	Multi-species biofilm	Downregulation of biofilm formation genes	[244]
Chitosanase	*Hermertia illucens*	*E. coli* and *Micrococcus flavus*	Moderate to high inhibitory activity	[245]
Cellulase	*Coptotermes ceylonicus*	*E. coli* and *B. subtills*	Effective inhibition	[246]
L-asparaginase	*Purpureocillium lilacinum*	*P. aeruginosa*, *K. pneumoniae*, *S. typhimurium*, *P. vulgaris*, *S. aureus*, and *L. monocytogenes*	Chitosan NAPs increased efficacy, high ZOI	[247]
Pectinase	*Nocardiopsis dasnonivelli*	*Bacillus* spp., *S. aureus*, *E. coli*, and *K. pneumoniae*	Limited antibacterial activity	[248]
Lipase	*Aspergillus niger*	MRSA, *P. mirabilis*, *P. aeruginosa*, and *E. coli*,	Distortion of cell shapes indicates metabolomic disturbances	[249]
Chitosanase	*Hermertia illucens*	*E. coli*, *S. aureus*, *P. aeruginosa*, and *B. subtilis*	Similar inhibitory activity with antibiotics tested	[250]
Lipase and protease	*P. mirabilis*	*S. aureus*, *P. aeruginosa*, *E. coli*, and *B. subtilis*	Inhibitory effect evident in ZOI	[251]
Lyase	*Flavobacterium multivorum*	*P. aeruginosa*	Biofilm dissolution	[252]
Phospholipase	*Bothrops erythromelas*	*E. coli*, *S. aureus*, and *A. baumannii*	Antibacterial and antibiofilm activities	[253]

## Data Availability

No new data were created or analysed in this study. Data sharing is not applicable to this article.
